# Overview of particulate air pollution and human health in China: Evidence, challenges, and opportunities

**DOI:** 10.1016/j.xinn.2022.100312

**Published:** 2022-09-06

**Authors:** Qingli Zhang, Xia Meng, Su Shi, Lena Kan, Renjie Chen, Haidong Kan

**Affiliations:** 1 School of Public Health, Key Lab of Public Health Safety of the Ministry of Education and NHC Key Lab of Health Technology Assessment, Shanghai Institute of Infectious Disease and Biosecurity, Fudan University, Shanghai 200032, China; 2 Bloomberg School of Public Health, Johns Hopkins University, Baltimore, Maryland, MD 21205, USA; 3 Children’s Hospital of Fudan University, National Center for Children’s Health, Shanghai 201102, China

## Abstract

Ambient particulate matter (PM) pollution in China continues to be a major public health challenge. With the release of the new WHO air quality guidelines in 2021, there is an urgent need for China to contemplate a revision of air quality standards (AQS). In the recent decade, there has been an increase in epidemiological studies on PM in China. A comprehensive evaluation of such epidemiological evidence among the Chinese population is central for revision of the AQS in China and in other developing countries with similar air pollution problems. We thus conducted a systematic review on the epidemiological literature of PM published in the recent decade. In summary, we identified the following: (1) short-term and long-term PM exposure increase mortality and morbidity risk without a discernible threshold, suggesting the necessity for continuous improvement in air quality; (2) the magnitude of long-term associations with mortality observed in China are comparable with those in developed countries, whereas the magnitude of short-term associations are appreciably smaller; (3) governmental clean air policies and personalized mitigation measures are potentially effective in protecting public and individual health, but need to be validated using mortality or morbidity outcomes; (4) particles of smaller size range and those originating from fossil fuel combustion appear to show larger relative health risks; and (5) molecular epidemiological studies provide evidence for the biological plausibility and mechanisms underlying the hazardous effects of PM. This updated review may serve as an epidemiological basis for China’s AQS revision and proposes several perspectives in designing future health studies.

## Public summary

• Acute effects of PM are smaller in China compared with developed countries
• Health effects caused by PM depend on particle composition, source, and size
• There are no thresholds for the health effects of PM
• Mechanistic studies support the biological plausibility of PM’s health effects

## Graphical abstract

**Figure:**
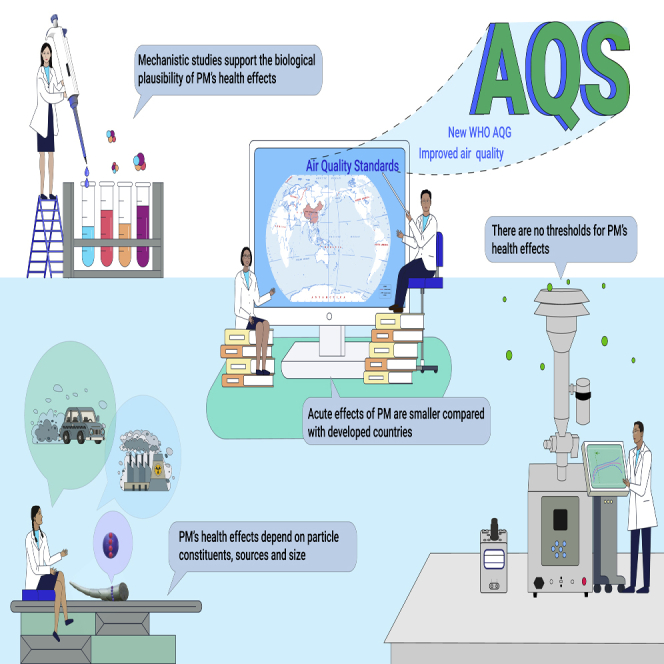

## Introduction

Rapid economic development over the past few decades in China resulted in drastic increases in the emission of air pollutants. Although there has been continuous air quality improvement following a series of stringent control policies, air pollution is still an important public health threat in China. The Global Burden of Disease Study estimated that in 2019, air pollution was responsible for 1.85 million deaths in China, among which 1.42 million were attributable to particulate matter (PM).^1^

In 2021, World Health Organization (WHO) tightened the global Air Quality Guidelines (AQG), for both fine particulate matter (particulate matter with an aerodynamic diameter ≤ 2.5 μm, PM_2.5_) and inhalable particles (particulate matter with an aerodynamic diameter ≤ 10 μm, PM_10_) due to ample evidence of adverse health effects even at very low concentrations of PM. It was estimated that 1,215,000 premature deaths would have been avoided in China by achieving the new WHO AQG target in comparison with the PM_2.5_ level in 2020.^2^ The current Chinese Air Quality Standards were made with references to the interim targets of WHO AQG 2005. The release of the new WHO AQG may stimulate the Chinese government to consider a new round of standard revision. A comprehensive evaluation of epidemiological evidence on health effects of air pollution among the Chinese population is central to revise air quality standards (AQS) in China and other developing countries experiencing apparent air quality problems.

During the last decade, air quality problems have led to widespread concerns in China. To support investigations on the health effects of air pollution and their toxicological mechanisms, the Chinese government has funded a number of projects, mainly including the National Key Research and Development Program titled “The Cause and Control Technology of Air Pollution” and the Major Research Program of the National Natural Science Foundation titled “Toxicology and Health Effects of Fine Particulate Matter.” With substantial financial support, scientific evidence on the health effects of air pollution, especially PM, has been rising rapidly in China in recent years. Compared with earlier studies that were largely ecological in nature,^3^ studies published in the recent decade were of higher quality in breadth, depth, and causality inference. A timely and systemic summary of these scientific evidences is key to revise China’s AQS and is informative in designing future health studies and air quality improvement projects worldwide.

We aimed to provide a comprehensive review on the up-to-date epidemiological evidence of PM and human health in China published during the last decade. This systemic review will include the following sections: (1) characteristics of PM air pollution in China; (2) epidemiological evidence on short-term and long-term associations between PM and human health; (3) findings from experimental or quasi-experimental studies; (4) differential health effects of PM constituents, sources, and size fractions; (5) biological mechanisms found in epidemiological studies of PM; (6) considerations in revising China’s AQS; and (7) conclusions, significance, and perspectives from the study.

## Levels and trends of particulate air pollution in China

Rapid economic development and rigorous environmental protection actions have driven dramatic shifts in China’s air pollution pattern over the past decades. Due to the dominant use of coal to generate energy, air pollution in China was once primarily characterized by coal smoke. Traffic-related air pollution (TRAP) is now prominent due to the fast urbanization of China and the growing number of motor vehicles. Consequently, air pollution in China has gradually changed into a complex pattern with coal smoke, TRAP, and secondary aerosols of similar importance.

**Figure 1:**
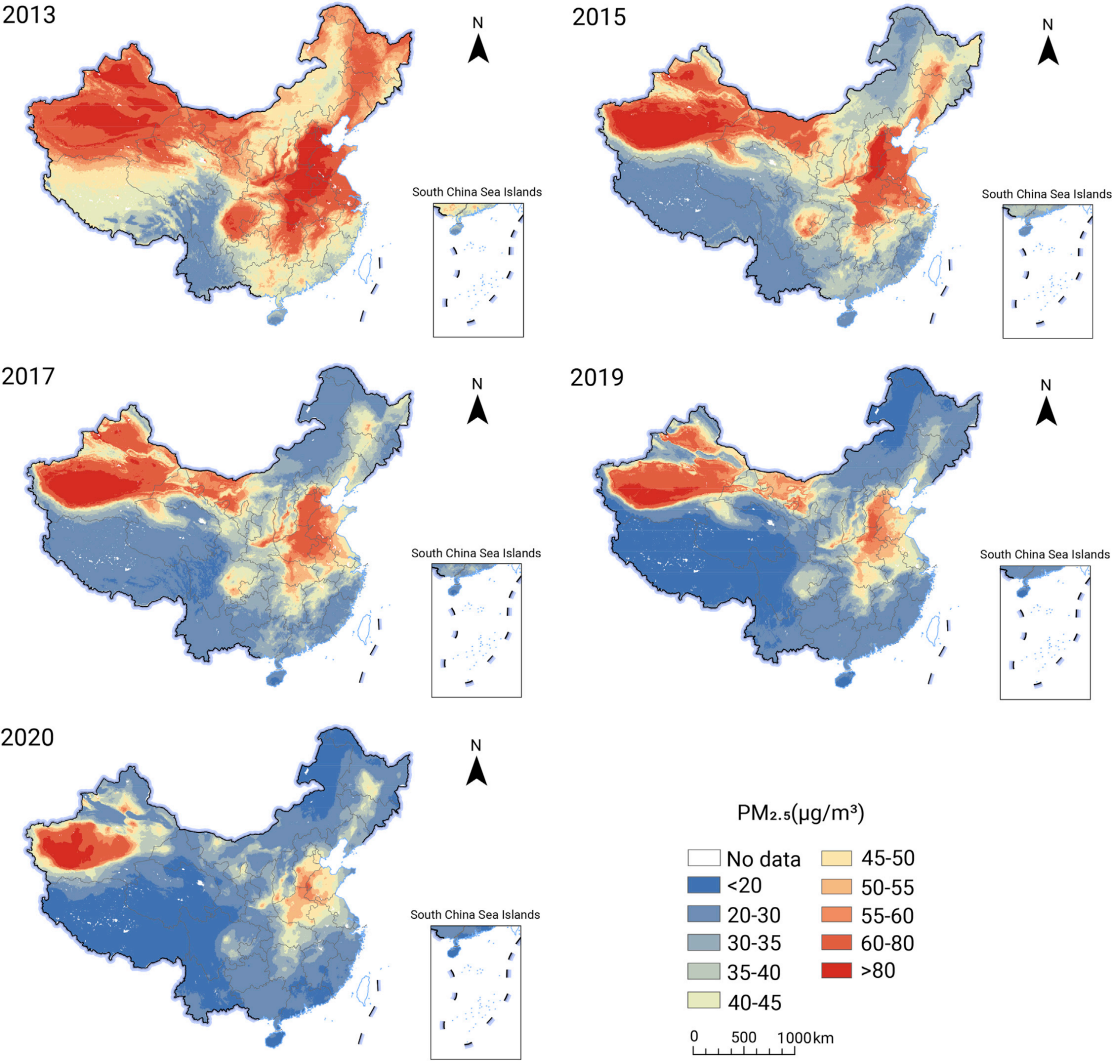
Annual average concentration of PM_2.5_ in China from 2013 to 2020

**Figure 2:**
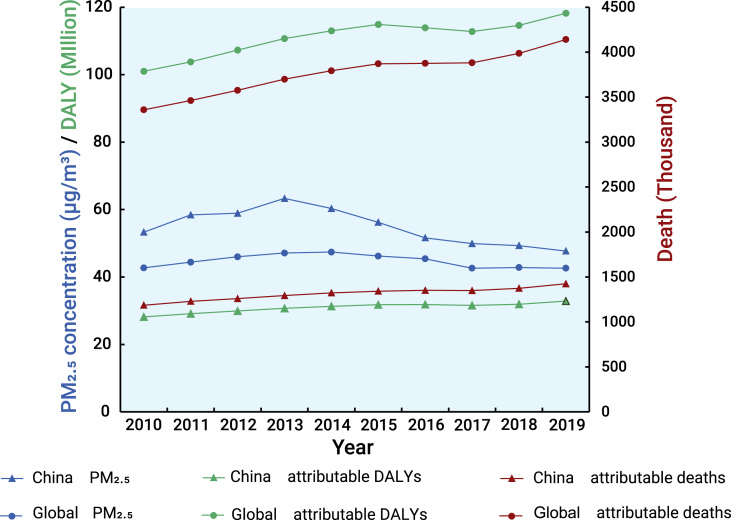
Average annual population-weighted PM_2.5_, number of deaths and DALYs attributable to PM_2.5_ in China compared with global data during 2010–2019 (data source: Global Burden of Disease Study 2019) (DALY, disability adjusted life year)

In the past decade, the Chinese government has implemented a series of rigorous policies and measures for air pollution control, such as the Air Pollution Prevention and Control Action Plan (2013–2017, referred to as CAP for short) and the Three-Year Action Plan to Win the Battle for a Blue Sky (2018–2020). Substantial improvements in air quality have been achieved following these actions (Figure 1). As shown in Figure 2, the annual average population-weighted PM_2.5_ concentrations have started to decline remarkably since 2013, but were still well beyond the global means. The annual average concentrations of PM_2.5_ and PM_10_ were 30 μg/m^3^ and 54 μg/m^3^ in 339 major Chinese cities in 2021, which are slightly lower than the current China AQS (35 μg/m^3^ for PM_2.5_ and 70 μg/m^3^ for PM_10_) but remain significantly higher than levels in the updated WHO AQG (5 μg/m^3^ for PM_2.5_ and 15 μg/m^3^ for PM_10_). The number of premature deaths and disability adjusted life years attributable to PM_2.5_ in China continues to increase, although this increasing trend is relatively modest compared with the global average (Figure 2).^4^

## Epidemiological evidence on health impacts of particulates in China

### Short-term associations

Short-term health effects typically refer to changes of clinical or subclinical outcomes triggered or induced by exposures that last within a few weeks. There has been a number of time-series, case-crossover, and panel studies examining the associations of short-term exposure to PM with a wide range of mortality, morbidity, and subclinical outcomes in China. China included PM_2.5_ in AQS for the first time in 2012 and established the National Urban Air Quality Real-time Publishing Plat-form (http://106.37.208.233:20035/) in 2013, which provided a unique opportunity to conduct large, multi-city studies. These multi-city studies provided more reliable evidence for the health effects of PM and more representative exposure-response (E-R) curves in Chinese populations.

#### Mortality

Mortality is the most severe and definitive health endpoint. The associations of short-term PM exposure with daily mortality have been extensively investigated in China. The associations between daily mean PM levels and daily aggregate deaths were usually examined using a time-series or case-crossover approach. Data on daily PM concentrations were generally collected from fixed-site monitoring stations, and daily mortality data were obtained from the Center for Disease Control and Prevention.

Emerging multi-city studies have demonstrated that short-term exposure to PM is significantly associated with increased mortality from all-natural causes and main cardiopulmonary diseases. Using time-series data in 38 of China’s largest cities (sampled population >200 million), Yin et al. found a 10-μg/m^3^ increase in concurrent day PM_10_ concentrations to be associated with a 0.44% increase in daily mortality, and the impact was greater on cardiorespiratory mortality (0.62%) than non-cardiorespiratory mortality (0.26%).^5^ Using data from 250 counties, Sun et al. conducted a time-series study and reported that heavy PM_2.5_ pollution events had independent effects on daily mortality from all-cause, nonaccidental, circulatory, and respiratory diseases.^6^ Using the largest nationwide data in 272 Chinese cities,^7^ Chen et al. reported that a 10-μg/m^3^ increment in a 2-day moving average (lag 01 days) of PM_2.5_ was associated with increases of 0.22% from all-cause mortality, 0.27% from cardiovascular mortality, and 0.29% from respiratory mortality. The authors also observed an apparent plateauing trend in the E-R curves at high concentrations of PM_2.5_. Another nationwide time-series study in 267 Chinese cities reported higher mortality risks (0.44% for all-cause mortality, 0.59% for respiratory mortality, and 0.50% for cardiovascular mortality) per 10-μg/m^3^ increase in time-weighted average PM_2.5_ exposures.^8^ This study indicated that accounting for indoor exposure may help improve the estimation of PM_2·5_-associated mortality. Multi-city studies in China generally reported a 0.22%–0.44% increase in all-cause mortality, 0.27%–0.62% increase in cardiovascular mortality, and 0.29%–0.59% increase in respiratory mortality per 10 μg/m^3^ increase in daily PM concentrations. The coefficients of E-R relationships were appreciably smaller than those reported in Europe and North America. For example, a meta-analysis including 68 time-series studies reported that the pooled estimates per 10-μg/m^3^ increase in PM_2.5_ were 1.23%, 3.81%, and 2.26% in Europe, and 0.94%, 1.39%, and 0.84% in North America for all-cause, respiratory, and cardiovascular mortality, respectively.^9^

Compared with studies focusing on mortality of the general population, few studies in China have examined the association between PM and child deaths. A recent national case-crossover study including 61,464 under-5 mortality cases found that short-term exposure to PM_2.5_ was significantly associated with under-5 mortality from all natural causes as well as from specific diseases such as preterm birth, diarrhea, pneumonia, and digestive diseases. The magnitude of association is considerably larger than that estimated in the general population, suggesting the notable susceptibility among children to the acute effects of PM.^10^

#### Morbidity

Compared with mortality, morbidity might be more sensitive to reflect the acute health effects of air pollution. There are dozens of time-series or case-crossover studies examining the short-term associations of daily PM levels measured at fixed-site monitors with daily hospital admissions, outpatient visits, or emergency room visits.

Most past studies focused on the short-term associations with respiratory and cardiovascular morbidity. Using time-series data from 21 cities in southwestern China, Qiu et al. found a 1.52% increase in hospitalizations for respiratory diseases per 10-μg/m^3^ increase in coarse particles.^11^ Gu et al. conducted a time-series study to assess the association between air pollution and daily hospital admissions in 252 Chinese cities using a national registration database of electronic inpatient discharge records from the Hospital Quality Monitoring System (HQMS). The authors reported that a 10-μg/m^3^ increase in same-day PM_2.5_ was associated with a 0.29% increase in daily hospital admissions of respiratory diseases.^12^ In another time-series analysis using the HQMS data covering 248 Chinese cities, the authors found that a 10-μg/m^3^ increase in PM_2.5_ was associated with increases of 0.19%, 0.26%, and 0.26% for the same-day hospital admissions of total cerebrovascular disease, ischemic stroke, and transient ischemic attack, respectively.^13^ Similarly, Tian et al. conducted a time-series study based on the Urban Employee Basic Medical Insurance (UEBMI) in 184 major Chinese cities and found short-term exposure to PM_2.5_ to be associated with increased hospital admissions of various cardiovascular diseases (CVDs). A 10-μg/m^3^ increase in PM_2.5_ was associated with increased hospital admission due to overall CVD (0.26%), ischemic heart disease (0.31%), heart failure (0.27%), heart rhythm disturbances (0.29%), and ischemic stroke (0.29%) on the concurrent day. The magnitudes of association for the two nationwide time-series studies were generally comparable and both observed a plateauing pattern at higher PM_2.5_ levels in the E-R relationship curves.^13^^,^^14^ Overall, multi-city studies in China observed 0.29%–1.52% increases in respiratory hospital admissions and 0.19%–0.26% increases in cardiovascular hospital admissions per 10-μg/m^3^ increase in PM. However, a meta-analysis including 54 time-series studies of PM_2.5_ and hospital admissions found higher estimates in Europe (0.91% in cardiovascular hospital admissions and 1.90% in respiratory hospital admissions).^9^

PM has recently been associated with mental disorders, which is becoming one of the major contributors to disease burden. A nationwide time-series study in 252 Chinese cities based on data from HQMS reported that each 10-μg/m^3^ increase in PM_2.5_ was associated with 0.21% increase in hospital admissions for mental and behavioral disorders on the concurrent day. Using data from the UEBMI and the Urban Resident-based Basic Medical Insurance (URBMI) database, Gu et al. conducted a time-series analysis in 75 cities. They found a 0.52% increase in depression hospital admissions per 10-μg/m^3^ increase in PM_2.5_.^15^ In a case-crossover study in 26 Chinese cities, Wang et al. also reported a positive association between short-term PM exposure and the number of hospital admissions for depression (2.92% and 3.55% increases per interquartile range (IQR) change of PM_2.5_ and PM_10_, respectively).^16^ In a time-series study using the data of 56 cities from UEBMI and URBMI database, Ma et al. found that daily hospital admissions for anxiety were only marginally significantly associated with PM_2.5_.^17^

In addition, other health morbidities have also been associated with short-term PM exposure. These include diseases from endocrinal, nutritional, metabolic, nervous, digestive, musculoskeletal, or genitourinary systems.^12^^,^^18^^,^^19^

#### Subclinical outcomes

Subclinical changes can occur earlier than the presentation of mortality or a morbidity event, so they are more sensitive to short-term PM exposure. This type of evidence is very helpful to the early prevention of PM-related diseases as well as the establishment of biological plausibility underlying the observed associations of PM with mortality and morbidity. A number of observational studies, mainly panel studies, have demonstrated significant associations of short-term PM exposure with changes in a wide range of subclinical outcomes, including fractional exhaled nitric oxide, lung function, blood pressure, heart rate variability (HRV), renal function, and various biomarkers in biospecimens of healthy adults,^20^, ^21^, ^22^, ^23^ older individuals,^24^ children,^25^ and cardiopulmonary disease patients.^26^, ^27^, ^28^, ^29^, ^30^, ^31^, ^32^ Moreover, several short-term intervention studies suggested that reducing air pollution exposure by personal protective measures (air purifiers and particulate-filtering respirators) or air quality improvement actions can help alleviate various subclinical effects induced by PM exposure.^33^, ^34^, ^35^, ^36^, ^37^ Apparent heterogeneity was observed for these findings, probably due to the vast differences in study designs, characteristics of air pollution mixture, population susceptibility, sample size, exposure assessment, outcome measurements (e.g., laboratory tests), statistical models, and covariates adjustment.

**Figure 3:**
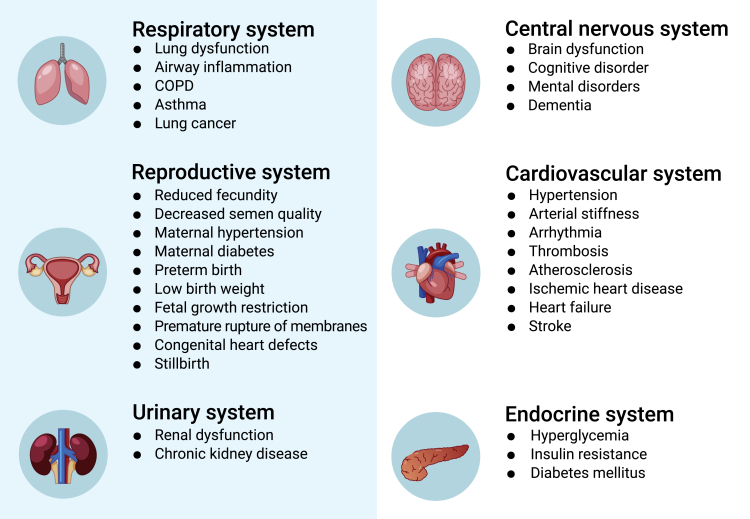
Main health outcomes of particulate air pollution summarized from epidemiological studies in China

In summary, ample epidemiological studies have provided largely consistent evidence for the associations between short-term PM exposure and increased risk of mortality and morbidity for a wide range of diseases, especially those of the cardiopulmonary system (Figure 3). The E-R relationship curves tend to be steeper at lower concentrations and plateaued at higher levels of PM without a discernible threshold. The magnitude of association seems to be larger among vulnerable populations, but somewhat smaller in China than in developed countries. Despite the ubiquitous heterogeneity in studies of subclinical effects, they still provided evidence on the coherence and biological plausibility for the hazardous effects of short-term PM exposures. The disparities in risk estimates between China and other countries might be attributable to differences in several areas. First, PM concentrations in China were several-fold higher than those in developed countries, and the E-R curves tended to be leveled off at higher PM concentrations. Second, the composition and sources that determine the PM toxicity also varied between China and other countries. For example, particles in China have higher contents in crustal materials, dust, and constituents from coal combustion, which may result in relatively lower toxicity than those in developed countries that mainly originated from traffic.^38^^,^^39^ Third, the age structure is relatively younger in China in comparison to developed countries, which might further decrease the population susceptibility to PM exposure.

### Long-term associations

Long-term associations refer to chronic health effects of exposures that last between several months and a few decades. The PM concentrations were often estimated by an exposure model. The long-term effects of PM were usually assessed using cross-sectional studies or cohort studies. Although cross-sectional studies have reported associations of annual PM exposures with multiple adverse health outcomes, these findings might be limited by causal inference. In contrast, cohort studies could provide reliable epidemiological evidence on the health effects of long-term exposure, constituting the basis for revising AQS and conducting health risk assessment. Dozens of cohort studies conducted in North America and Europe have demonstrated adverse health effects of long-term PM exposure at relatively low concentrations. However, it remains uncertain whether these associations found in developed countries are applicable to China, a country where PM concentrations are several-fold higher than those observed in North America and Europe.

#### Mortality

In the recent decade, several prospective cohort studies have emerged in China and have reported significant associations of long-term PM_2.5_ exposure with mortality from all causes or a range of cardiopulmonary diseases. In 2017, Yin et al. assessed the effect of long-term PM_2.5_ exposure on mortality risk using a nationwide prospective cohort among 189,793 Chinese men aged 40 years or more.^40^ In the Chinese Male Cohort, the hazard ratio (HR) of mortality per 10-μg/m^3^ increase in PM_2.5_ was 1.09 for nonaccidental causes, 1.09 for CVD, 1.12 for chronic obstructive pulmonary disease (COPD), and 1.12 for lung cancer. This study observed nonlinear relationships between PM_2.5_ and mortality outcomes with greater risks at higher concentrations. Similar estimates were obtained from another prospective cohort based on the Prediction for Atherosclerotic cardiovascular disease Risk in China (China-PAR) project. Among nearly 117 thousand participants aged ≥18 years old, the China-PAR project observed that each 10-μg/m^3^ increase in PM_2.5_ would increase risks of nonaccidental mortality (HR: 1.11), cardio-metabolic mortality (HR: 1.22),^41^ and CVD mortality (HR: 1.16).^42^ This cohort revealed a weak exponential E-R curve, which showed a steeper slope at higher PM_2.5_ concentrations. Based on the Chinese Longitudinal Healthy Longevity Survey (CLHLS) among 13,344 adults aged 65 years and older, another nationwide prospective cohort study estimated that a 10-μg/m^3^ increase in average 3-year PM_2.5_ exposure led to an increase in all-cause mortality (HR: 1.08) with a steeper E-R curve at lower concentrations.^43^ In addition to the aforementioned nationwide prospective studies, some cohort studies conducted in specific regions of China found significant associations with mortality from all-cause, stroke, cancer, renal failure, etc.^44^, ^45^, ^46^ In general, all of these cohort studies obtained relatively comparable estimates on the mortality effects of long-term PM_2.5_ exposure in China (approximate 10% increase in all-cause mortality per 10-μg/m^3^ increase in PM_2.5_), and also estimates comparable to those reported in developed countries.^47^^,^^48^

#### Prevalence and morbidity

Although many cross-sectional studies have found long-term associations between PM and prevalence of specific diseases in China, a limited number of Chinese cohort studies have explored the effects of long-term PM exposure on morbidity.

Chronic exposure to PM_2.5_ has been shown to be associated with increased risks of respiratory and cardiovascular diseases. Some cross-sectional studies indicated positive associations between PM_2.5_ and prevalence of chronic respiratory diseases, especially COPD.^49^^,^^50^ To our knowledge, no cohort study in mainland China has assessed the long-term effect of PM_2.5_ on incidence of respiratory diseases. Cohort studies based on the China-PAR project reported increased morbidity risks of CVD (HR: 1.25),^42^ coronary heart disease (HR: 1.43),^51^ and stroke (HR: 1.13) per 10-μg/m^3^ increase in PM_2.5_.^52^ Another cohort study using data from China Family Panel Studies (CFPS) also indicated higher CVD risk associated with long-term PM_2.5_ exposure, with an HR of 1.29 per IQR (27.9 μg/m^3^) increase of PM_2.5_.^53^

The increased risk of cancer incidence, especially lung cancer, has also been found to be associated with PM. Using data on incident lung cancer (N = 368,762) in 75 communities from the National Cancer Registration of China, Guo et al. conducted a prospective analysis and found that the relative risk of lung cancer incidence was 1.06 for men, and 1.15 for women per 10-μg/m^3^ increase in 2-year average PM_2.5_ concentration.^54^ Through a random 5% sample among UEBMI beneficiaries in China from 2013 to 2016, a total of 16,483 new lung cancer cases were identified from 12,966,137 insurance beneficiaries. Using this data, Zhang et al. observed that a 10-μg/m^3^ increase in a 3-year PM_2.5_ exposure was associated with a 12% increase in lung cancer risk.^55^ Using the China-PAR project, Li et al. found significantly higher lung cancer risk in participants exposed to the second to fifth quintiles of PM_2.5_ concentrations than those exposed to the first quintile.^44^

PM_2.5_ has been found to increase the morbidity risk of metabolic syndromes, such as diabetes, obesity, hypertension, and dyslipidemias. Cohort studies based on the China-PAR project suggested that a 10-μg/m^3^ increase in long-term PM_2.5_ exposure was associated with increments of 15.66% in diabetes,^56^ 13.5% in overweight/obesity,^57^ and 11.0% in hypertension.^58^ Some cross-sectional studies in China also supported a positive association between prevalence of metabolic syndrome and prolonged PM_2.5_ exposure.^59^^,^^60^

Mounting evidence from case-control studies, cross-sectional studies, and cohort studies (e.g., birth cohorts) has shown that long-term exposure to PM_2.5_ may impair maternal, reproductive and children’s health, and increase the risk of multiple adverse birth outcomes. Several studies in China have linked PM_2.5_ to reduced fecundity and decreased male semen quality.^61^, ^62^, ^63^ Some gestational diseases, such as hypertension^64^ and diabetes mellitus,^65^ were also observed to be associated with PM_2.5_. Moreover, prenatal PM_2.5_ exposure was reported to be responsible for various adverse birth outcomes, including preterm birth,^66^^,^^67^ low birth weight,^68^ fetal growth restriction,^69^ premature rupture of membranes,^70^ congenital heart defects,^71^ and even stillbirth.^72^ Additionally, PM_2.5_ exposure during pregnancy or early life was reported to impact the health status of the child during childhood, including increased risk of respiratory diseases,^73^ overweight or obesity,^74^ autism,^75^ and neurodevelopmental damage.^76^

#### Subclinical outcomes

Some cross-sectional and longitudinal studies have linked long-term exposure to PM to selected subclinical outcomes, including reduced lung function,^77^^,^^78^ and increased blood pressure,^79^^,^^80^ increased arterial stiffness,^81^ reduced renal function,^82^ poor cognitive function,^83^ increased level of blood glucose^84^ and lipids,^85^ worsening insulin resistance,^86^ elevated platelet counts,^87^ and so on. In particular, one recent study associated PM_2.5_ exposure with brain network dysfunction at multiple levels, presenting poorer reasoning and problem-solving skills as well as higher-trait anxiety/depression.^88^

In summary, there is sufficient evidence supporting the association between long-term PM exposure and increased mortality and morbidity for a wide range of diseases (Figure 3). Findings from nationwide prospective cohort studies demonstrated an approximate 10% increase in all-cause mortality per 10-μg/m^3^ increase in long-term PM_2.5_ exposure, levels which were generally comparable in magnitude to those reported in cohort studies conducted in North America and Europe.^47^^,^^48^ The adverse effects of long-term PM exposure on subclinical indicators provide some biological plausibility for the increase in mortality and morbidity observed in cohort studies.

### Experimental or quasi-experimental studies

Compared with observational studies, intervention studies are one of the optimal designs for inferring a causal relationship because intervention studies have a clear chronological order of exposure and events and can better control for confounders. In the last decade, several quasi-experimental studies at the population level have evaluated the health benefits of air pollution abatement due to clean air policies or large-scale events such as the Olympics. Moreover, some randomized, crossover trials at the individual level have been conducted to assess the potential protective effects of using indoor air purification, wearing face masks, and taking dietary supplements against air pollution. Findings from these intervention studies were valuable in inferring causality between air pollution and various health outcomes and in evaluating the effectiveness of exposure mitigation measures.

#### Quasi-experimental studies

China has promulgated a series of stringent clean air policies since 2013, including CAP (2013–2017), leading to dramatic improvements in annual air quality. During certain key prominent events, such as the Beijing Olympics and the Nanjing Youth Olympics, aggressive air pollution control actions were implemented in order to temporarily improve air quality. In 2003, the Health Effects Institute provided a conceptual framework of the “chain of accountability” describing how a regulatory action can impact emissions, pollutant concentrations, subject exposures, and human health.^89^ These policies and events in China offered unique opportunities to conduct “accountability” studies by evaluating whether the populations impacted by these policies received health benefits.

With the implementation of CAP, the annual average concentrations of PM_2.5_ in China decreased substantially from 67.4 μg/m^3^ in 2013 to 45.5 in 2017 μg/m^3^. By virtue of the overlapped periods between CAP and the China Health and Retirement Longitudinal Study (CHARLS), Xue et al. conducted a quasi-experimental study in a retrospective sample of ∼20,000 middle-aged and older adults in ∼150 county-level regions. During 2013 to 2017, the CHARLS project conducted three waves of surveys in 2011 (before CAP), 2013, and 2015 (after CAP). Using a difference-in-differences approach, the authors found significant associations of PM_2.5_ reduction with decreased risk of depression,^90^ improved blood lipid levels, and decreased risk of dyslipidemia,^91^ as well as reduced medical expenditure.^92^ Another quasi-experimental study was conducted based on the CLHLS among 2,812 participants aged 60 years and older from the 2014 and 2018 waves. In this study, 18 out of the 23 provinces set a target of reducing PM by at least 5% annually from 2014, while the remaining five provinces did not set a PM reduction target. Yao et al. found a significant smaller decline in cognitive function among individuals living in areas with a PM reduction target than those in areas without a target.^93^

During the 2008 Beijing Olympic Games, the Chinese government implemented strict measures to cut air pollution emissions, resulting in a reduction of 31% in PM_2.5_ and 35% in PM_10_ during the Olympic period compared with the non-Olympic period.^94^ Taking advantage of the quasi-experimental conditions, some epidemiological studies evaluated the potential health benefits of reducing air pollution. Su et al. found that air quality improvement during the 2008 Olympic Games led to reduction of CVD mortality risk in Beijing.^95^ Using data from a birth registry (83,672 term births to mothers), Rich et al. found short-term reductions of air pollution in the eighth month of gestation during the 2008 Olympic period to be associated with higher birth weight compared with pregnancies during the same dates in 2007 and 2009.^96^ In 125 healthy young adults, Rich et al. observed significant improvements in soluble P-selectin and C-reactive protein levels from the pre-Olympic period to the during-Olympic period, and increases in systolic blood pressure and P-selectin from the during-Olympic period to the post-Olympic period.^97^ In the same study population, Huang et al. observed large decreases in biomarkers of respiratory inflammation and respiratory and systemic oxidative stress from the pre-Olympic period to the during-Olympic period, while larger increases in the same biomarkers from the during-Olympic period to the post-Olympic period were observed.^98^ The lower air pollution level during the Olympics was also reported to be associated with decreased levels of systemic oxidative stress and respiratory inflammation in children.^99^^,^^100^

The 2014 Nanjing Youth Olympics was another opportunity to evaluate the health benefits of transient air quality improvement. Li et al. conducted a quasi-experimental study among 31 healthy adults and observed that some biomarkers of systemic inflammation significantly decreased from the pre-Olympic period to the Olympic period, while they increased significantly from the Olympic period to the post-Olympic period.^101^

Health impacts of translocation have been investigated, taking advantage of the different air pollution levels between areas. Lin et al. conducted a biomarker-based quasi-experimental study on 26 college students traveling between Los Angeles and Beijing.^102^ The investigators found that circulating levels of six lipid peroxidation biomarkers and two inflammatory biomarkers increased and the antioxidative activities decreased when participants traveled from Los Angeles to Beijing. These changes in proinflammatory and pro-oxidative biomarkers were reversed after the participants returned to Los Angeles. In the Healthy Volunteer Natural Relocation study, 41 college students who relocated from a suburban campus to an urban campus were enrolled and health indicators were repeatedly examined before and after the relocation. The authors found that PM_2.5_ was associated with a reduction in lung function,^103^ increases in blood pressure,^104^ and increases in certain inflammatory biomarkers.^105^

#### Personalized intervention studies

In addition to the governmental efforts to reduce air pollutant emissions, personalized mitigation measures are also helpful to decrease exposures to particulates and mitigate their harmful effects. Several intervention studies explored the short-term health benefits due to the use of air purifiers, masks, and dietary supplements. These trials, using a randomized controlled design, also shed light on the biological processes involving health effects from particulates.

The use of high-efficiency particulate air purifiers appears to be a promising tool to decrease personal exposure to PM as people typically spend most of their time indoors. Several randomized, double-blind, crossover studies have suggested potential health benefits in reducing indoor PM concentrations through air purification. Among 35 healthy college students from Shanghai, Chen et al. found that a 2-day use of air purification could result in a 57% reduction of PM_2.5_ exposures (from 96.2 to 41.3 μg/m^3^). This intervention was associated with significant decreases in blood pressure, airway inflammation, and circulating levels of inflammatory and thrombogenic biomarkers, as well as the increases of DNA methylation in repetitive elements and in genes of pro-inflammation, coagulation, and vasoconstriction.^34^^,^^106^ In another trial among 55 healthy college students in Shanghai, the 9-day average exposure levels of PM_2.5_ were 24.3 and 53.1 μg/m^3^ under real and sham purification, respectively. In this trial, Li et al. found significantly decreased levels of blood pressure, hormones, insulin resistance, oxidative stress, and inflammation among individuals with lower PM exposures. Metabolomics profiling further presented significant between-group differences in glucose, amino acids, fatty acids, and lipids and suggested the activation of hypothalamus-pituitary-adrenal (HPA) and sympathetic-adrenal-medullary axes by PM exposure.^33^ Higher PM_2.5_ was also associated with increases in proteins or mRNA expression implicated in inflammation and vasoconstriction, and reductions in miRNAs regulating expression of corresponding proteins or mRNA.^107^ Epigenome-wide association analysis based on the same trial has also shown altered methylation levels of annotated genes, which were involved in potential pathophysiological pathways of PM_2.5_ exposure.^108^ Several other intervention studies assessed the health impact of using air purifiers in seniors. For example, in a trial including 24 healthy residents of an aged-care center in Chongqing, 48-h use of air filtration was found to be associated with significantly decreased biomarkers of inflammation and coagulation.^109^ However, in a 2-week intervention among 35 non-smoking senior participants in Beijing, the investigators did not observe any improvements in most cardiorespiratory outcomes including lung function, blood pressure, and HRV.^110^

As a simple and practical intervention to reduce personal exposure to air pollution, especially outdoor air pollution, particle-filtration face masks or respirators have been reported to alleviate the harmful effects of air pollution among both healthy young adults^36^ and CVD patients in China.^111^ For example, in a randomized crossover trial among 24 healthy young adults in Shanghai, Shi et al. found that wearing particulate-filtering respirators for 48 h was associated with decreased blood pressure and increased HRV.^36^ In another trial among 40 healthy young adults in Beijing, wearing respirators for 4 h in the underground subway was associated with increases in most HRV parameters, and decreases in ST segment elevation and heart rate.^35^ In a larger trial involving 98 patients with coronary heart disease, Langrish et al. found the use of a highly efficient face mask during a 2-h walk along a city center route in Beijing to be associated with decreased self-reported symptoms and improved cardiovascular measures including reduced maximal ST segment depression, decreased mean arterial pressure, and increased HRV.^111^ In a trial involving 15 healthy young adults who walked along a busy-traffic road for 2 h in Beijing, results showed that wearing face masks could lead to a reduction in airway inflammation.^112^

Oxidative stress is widely considered to be one of the predominant pathways underlying the adverse health effects induced by air pollution, so it is biologically plausible that dietary supplementation with antioxidants may mitigate the harmful effects of air pollution. In a randomized, double-blind, placebo-controlled trial among 65 healthy college students in Shanghai, fish-oil supplementation presented possible cardiovascular health benefits by alleviating PM_2.5_-mediated systemic inflammation, coagulation, endothelial function, oxidative stress, and neuroendocrine stress response^113^ as well as promoting skin health benefits by mitigating PM_2.5_-mediated skin inflammation and oxidative stress.^114^ Another randomized, double-blind, placebo-controlled trial among 118 adults with elevated blood pressure in Beijing evaluated the protective effects of L-arginine supplementation based on a 2-h walk along a busy road. The results suggested that the supplementation with L-arginine may reduce the adverse effects of air pollution on blood pressure and platelet mtDNA methylation.^115^^,^^116^

In summary, existing Chinese studies provide robust evidence on the favorable short-term changes of a range of health outcomes due to personalized intervention behaviors and air pollution abatement policies. However, very few experimental or quasi-experimental studies evaluated long-term health benefits (especially on clinical events). Therefore, it remains to be determined whether these favorable molecular or subclinical changes reported can be translated into clinical benefits (e.g., reduced mortality and morbidity).

### Differential health effects of particulate matter constituents, sources, and size fractions

Most epidemiological studies on PM focused on the health effects of total mass concentrations. However, the toxicity of PM was determined by features related to chemical constituents, sources, and size fractions. Identifying the physicochemical characteristics that have critical toxicity and health effects is important to conduct accurate health risk assessments, develop targeted air quality management strategies, and tailor localized public health responses.

PM_2.5_ is a mixture comprising a complex chemical composition, including organic carbon, inorganic carbon, inorganic ions, and metallic or non-metallic elements, each of which might have independent or interactive effects with each other. Because the components are closely related to specific sources, understanding the relative importance of various constituents is of great significance for identifying the more harmful sources. Some epidemiological studies in China have attempted to identify key constituents responsible for PM_2.5_-related health effects. Using data from Shanghai Health Insurance Bureau, Qiao et al. performed a time-series study and reported that organic carbon and elemental carbon have robust associations with daily emergency room visits. In a panel study of 40 college students in Beijing, Wu et al. found that short-term exposure to carbonaceous fractions, some ions and metals were closely related to increased blood pressure,^104^ and certain metallic constituents may induce impairment in lung function,^117^ alterations in circulating biomarkers of endothelial function,^118^ and oxidative stress.^119^ In a panel study among 30 COPD patients in Shanghai, Chen et al. found organic carbon, elemental carbon, nitrate, and ammonium to be significantly associated with increased airway inflammation and decreased DNA methylation of encoding genes.^32^ Several cohort studies have also reported the long-term effects of specific constituents. For example, in a prospective analysis of 90,672 adults across 161 districts/counties in China, black carbon, organic matter, nitrate, ammonium, and sulfate had appreciably larger HRs of mortality from CVD and its subtypes than PM_2.5_ total mass, while soil dust had no increased risk.^120^ Another nationwide cohort of 14,331 adults (in CFPS) has demonstrated that black carbon, nitrate, ammonium, and sulfate were mainly responsible for the association between long-term exposure to PM_2.5_ and increased risks of total CVD and hypertension incidence.^53^ Using data from a nationwide birth cohort of 3,723,169 live singleton births in 336 Chinese cities, He et al. found organic carbon and black carbon to be associated with higher preterm birth risk.^121^ Although the results are not always consistent, transition metals, carbonaceous fractions, nitrates, and sulfates are likely to be responsible for the adverse effects of PM, suggesting that sources from fossil fuel combustion might be major contributors to adverse health effects.

Size fraction is another important feature to determine the health effects of PM. It is assumed that PM of a smaller size has higher toxicity as these particles have larger surface area, higher reactivity, and are more likely to penetrate deeper into the respiratory system. Moreover, the nano-scale particles might even be able to be absorbed into the blood after inhalation. Unlike PM_2.5_ and PM_10_, particulates of a smaller size were not criteria pollutants, and their health effects were not commonly investigated because of the lack of environmental monitoring data. As a major mass contributor to PM_2.5_ (over 80% of PM_2.5_ in China), PM_1_ (particulate matter with aerodynamic diameter ≤1 μm) has recently been reported to be related to various adverse outcomes, including cardiovascular morbidity and mortality,^122^^,^^123^ hypertension,^124^ asthmatic symptoms,^125^ metabolic syndrome,^60^ autism,^75^ and preterm birth.^126^ The health effects of PM_1_ were further found to be independent of other air pollutants^127^^,^^128^ and may be more harmful than PM_2.5_.^122^^,^^123^ PM also contains a large number of nano-scale particles, such as those <0.1 μm (i.e., ultrafine particles [UFPs]). Although these particles account for only a very small mass fraction of PM_2.5_, they contribute to the largest proportion of number concentrations. Recent evidence from time-series or panel studies conducted in megacities in China indicated that short-term exposure to UFPs was associated with a series of cardiopulmonary diseases or their biomarkers.^129^, ^130^, ^131^, ^132^, ^133^ Some studies in China further indicated that the detrimental effects of UFPs were independent of other air pollutants^132^ and stronger than larger particles.^132^^,^^133^ The WHO provided a qualitative statement of UFPs in 2021 for the first time, but did not conclude a particular AQG for UFPs due to insufficient epidemiological evidence.^134^

In summary, although increasing epidemiological studies evaluated the associations of constituent-specific and size-segregated particles with a variety of health outcomes in China, no consensus was reached on the specific chemical compositions or size ranges with predominant health hazards. A better understanding of the potentially differential health effects from various particle sources is of great value to develop more targeted policies to reduce air pollutant emissions. However, there is a lack of epidemiological evidence directly linking different particle sources to adverse health outcomes.

## An overview of human-based findings on biological mechanisms

**Figure 4:**
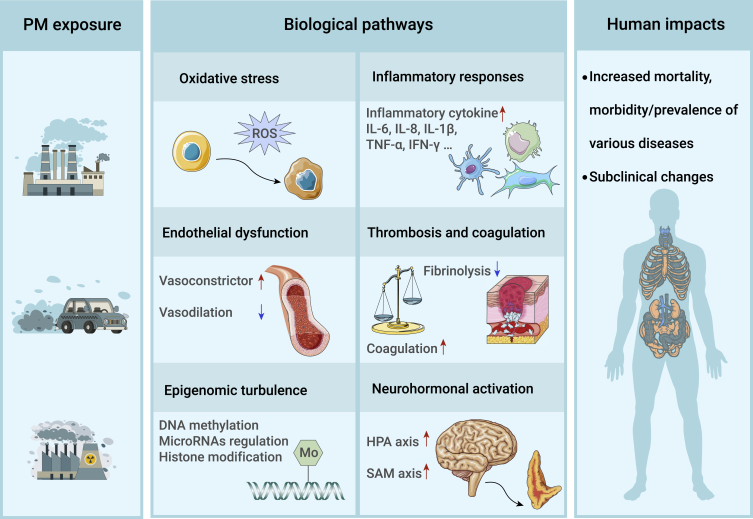
Biological pathways underlying the health effects of particulate air pollution proposed in molecular epidemiological studies of China Abbreviation: PM, particulate matter; ROS, reactive oxygen species; HPA axis: hypothalamus-pituitary-adrenal axis; SAM, sympathetic-adrenal-medullary axis.

Human-based mechanistic evidence is valuable for inferring causality as it provides biological plausibility. It is also helpful in identifying the early-effect biomarkers and the key physiopathological pathways that may be used in the prevention and intervention of PM-related diseases. A number of observational, experimental, and quasi-experimental studies in China have extensively explored the biological mechanisms underlying PM-related effects in various subpopulations. Biomarkers implicated in oxidative stress,^33^^,^^98^^,^^102^^,^^135^^,^^136^ inflammatory response,^20^^,^^23^^,^^32^^,^^97^^,^^107^^,^^137^, ^138^, ^139^ endothelial dysfunction,^107^^,^^140^ thrombosis and coagulation,^34^^,^^107^^,^^109^^,^^141^^,^^142^ and epigenetic changes^106^^,^^107^^,^^143^^,^^144^ were frequently reported to be associated with changes in PM exposures (Figure 4). These biomarkers were mostly examined in studies of short-term exposures, which still need to be confirmed by prospective cohort studies. Although most of these biomarkers or proposed pathways were not novel in the literature, the molecular epidemiological findings in China replicated mechanistic evidence in a highly polluted background.

With the development of high-throughput techniques, emerging studies in China investigated the biological mechanisms of PM using omics, such as genomics,^88^ epigenomics,^108^^,^^145^^,^^146^ transcriptomics, metabolomics,^33^^,^^147^, ^148^, ^149^, ^150^ lipidomics,^67^ and microbiomics.^20^^,^^151^, ^152^, ^153^, ^154^, ^155^ An omics analysis allows a hypothesis-free assessment of potential biological mechanisms, which is helpful to discover novel biological pathways and biomarkers. For example, in a randomized, double-blind, crossover trial among 55 healthy college students by Li et al., the metabolomic analysis identified a range of metabolic changes indicative of activations of the HPA and sympathetic-adrenal-medullary axes after short-term exposure to PM_2.5_. This novel finding added new mechanistic evidence for understanding the health effects of PM_2.5_.^33^ Other single-omics analyses revealed the alterations of a broad spectrum of biomolecules and unveiled some biological pathways triggered by air pollution.^107^^,^^148^^,^^156^ However, very few investigations explored the systemic alterations induced by air pollution using the multi-omics approach. In a randomized crossover trial of TRAP, the transcriptomics, proteomics, metabolomics, and lipidomics were incorporated simultaneously, and the changes in gene expression, proteins, metabolites, and lipids in response to short-term exposure to TRAP were mapped. The authors found that dozens of regulatory pathways were activated after TRAP exposure including some well-known pathways related to air pollution, such as inflammation, oxidative stress, coagulation, endothelin-1 signaling, renin-angiotensin signaling, and lipid metabolism, as well as certain novel pathways such as growth hormone signaling, adrenomedullin signaling, arachidonic acid metabolism, and vascular smooth muscle cell proliferation.^157^^,^^158^

It should be noted that most available mechanistic evidence in China was derived from observational studies or intervention studies conducted in real-word environments, which may, to some extent, be subject to residual confounding. In addition, almost all mechanistic studies focused on the short-term effects of PM, while the long-term effects were rarely investigated. More controlled-exposure trials and prospective cohort studies are warranted to elucidate the biological mechanisms underlying the health effects. A multiple-omics approach, especially from mediation or “meet-in-the middle” analysis, is encouraged to understand the global molecular changes and their causality relations more comprehensively in the systemic response induced by PM. Multiple cross-validation is also needed to improve the reliability and reproducibility of the findings.

## Considerations in revising China’s air quality standards

AQS is the cornerstone of air quality management. In China, it is the Ministry of Ecology and Environment’s responsibility to update the AQS regularly. The current China’s AQS (GB 3095-2012) was issued in 2012 with certain amendments made in 2018. Whether and when to start a new round of standard revisions has been put on the agenda of the Ministry of Ecology and Environment. The release of the new WHO AQG has inevitably triggered more discussions. As the WHO recommended, governments across the world, including China, can use the AQG in different ways depending on the local technical capabilities, economic capacity, air quality management policies, and other political and social factors.

Scientific evidence on the health effects of air pollution is essentially the cornerstone in setting/revising the AQS. In updating China’s AQS, the state-of-the-art science of the association between air pollution and health should be fully considered, with special emphasis on Chinese epidemiological data, whenever available. During the process of revising AQS, an extensive assessment of the best available evidence on E-R relationship is indispensable. Some factors, like population characteristics, climate, and air pollution level and sources, may modify the E-R relationship. For example, the acute health risks observed among the Chinese population are somewhat smaller in magnitude, per amount of pollution, than the risks found in developed countries. Therefore, local evidence is particularly important in revising China’s AQS.

In the meantime, revising China’s AQS simply based on epidemiological evidence might uncover that the intended standards are not achievable in practice. Facing the reality of relatively high air pollution levels and the demand for rapid economic development, it might be practical to set China’s AQS for major air pollutants using interim targets. Given the large disparities in air pollution levels and E-R relationships across China’s various regions, applying regional AQS should be encouraged. Also, the issue of environmental justice and social equity should be considered in the development of China’s AQS. For instance, the Chinese population is rapidly aging, and so China’s AQS should protect not only the general population, but also vulnerable subgroups such as those with chronic conditions (e.g., respiratory or cardiovascular diseases) and older people. Nevertheless, more in-depth research and discussion will be needed in order to have a balance between the ideal target value and practical feasibility in China.

## Conclusions, significance, and future perspectives

During the last decade, epidemiological studies on particulate air pollution and human health have been growing in China, comprising large, multi-city studies, prospective cohort studies, experimental and quasi-experimental studies, and molecular epidemiological studies. These studies have provided convincing evidence that particulate air pollution is associated with a wide range of adverse health effects, especially on the cardiopulmonary system (Table 1, graphical abstract). According to this comprehensive review on epidemiological studies conducted in China during the last decade, we can derive the following conclusions. Specifically, (1) short-term and long-term PM exposure increase the mortality and morbidity risk without a discernible threshold; (2) the magnitude of long-term associations with mortality observed in China were comparable with those in developed countries, whereas the magnitude of short-term associations were appreciably smaller; (3) governmental clean air policies and personalized mitigation measures are potentially effective in protecting public and individual health, but need to be confirmed using mortality or morbidity outcomes; (4) particles of smaller size range and those originating from fossil fuel combustion appear to show larger relative health risks; and (5) molecular epidemiological studies provide evidence for the biological plausibility and mechanisms underlying hazardous effects of PM.

**Table 1:** Overall summary of epidemiological evidence on the health effects of fine particulate air pollution in China

	Short-term (hours to days)			Long-term (months to years)		
	Number	Quality	Consistency	Number	Quality	Consistency
**Mortality**						
All-cause	+++	+++	+++	++	+++	+++
Respiratory disease	+++	+++	+++	++	++	+++
Cardiovascular disease	+++	+++	+++	++	++	+++
**Morbidity**						
Respiratory disease	+	++	++	++	+	+++
Cardiovascular disease	++	++	+++	+++	++	+++
Mental disorders	+	++	++	++	++	+++
Lung cancer	–	–	–	++	+++	+++
Metabolic syndrome	–	–	–	++	++	+++
Adverse reproductive outcomes	–	–	–	+++	+++	++
Chronic kidney disease	–	–	–	+	+	++
**Subclinical outcomes**						
Reduced lung function	+++	++	++	++	++	+++
Increased blood pressure	+++	+++	++	++	+	+++
Reduced heart rate variability	+++	+++	++	–	–	–
Increased arterial stiffness	–	–	–	+	+	+
Reduced renal function	–	–	–	+	+	+
Poor cognitive function	–	–	–	+	++	++
Increased blood glucose	–	–	–	+	+	+
Increased blood lipids	–	–	–	+	++	++
Insulin resistance	–	–	–	+	++	++

Number: +++ means many relevant studies (>10), ++ means several relevant studies (3–10), + means only 1 to 2 relevant studies.

Quality: +++ high (≥3 trial/quasi-experimental study/multi-cities studies for short-term effects, or ≥3 cohort studies for long-term effects), ++ moderate (1–2 trial/quasi-experimental study/multi-cities studies for short-term effects, or 1 to 2 cohort studies for long-term effects), + low (all studies are observational in nature and no multi-cities studies).

Consistency: +++ almost completely consistent, ++ partly consistent, + completely inconsistent.

–, no relevant studies.

These findings have implications for policy formulations and further investigations. First, the adverse effects of PM_2.5_ appear to have no discernable “safe” threshold, which indicated the necessity of the continuous improvement of China’s air quality. Second, the E-R association was found to vary by geographical area, which indicates that local specific conditions should be considered in future AQS revision, risk assessment, disease burden estimation, and policy formulation. Third, individuals with chronic conditions (e.g., respiratory or cardiovascular diseases), children, and older people were found to be vulnerable to the impacts of PM. This revelation has implications to protect sensitive populations and explore the mechanisms underlying the susceptibility. Finally, PM appears to adversely affect various human systems, which has enhanced the understanding of the spectrum of diseases affected by PM. Epidemiological and toxicological studies are warranted to further clarify their causal relationships in the future.

Although the adverse health effects of PM have been well documented in China, several questions or challenges remain to be solved in human-based studies, especially for stronger causality inference. Firstly, a cohort study is one of the optimal designs to infer a causal relationship because it could observe the natural process from long-term air pollution exposure to the incidence of adverse health outcomes. Evidence from cohort studies plays a crucial role in the formulation of AQS and assessment of health risks or disease burden. Although a large number of cohort studies conducted in developed countries have reported various hazards of long-term PM exposures, it remains uncertain whether these associations are also applicable in China. Although some recent prospective cohort studies in China have examined the long-term effects of PM exposure on total and cardiopulmonary mortality or morbidity, evidence from cohort studies is still lacking for examining mortality and morbidity due to a full spectrum of diseases. Secondly, accountability studies that evaluate long-term health benefits of clean air policies and intervention studies that reduce individual’s exposure are also needed to clarify the termination effects of air pollution. The applications of the difference-in-differences approach and the randomized crossover design have been shown to further improve the causality. Thirdly, controlled-exposure human trials are particularly useful to establish the biological causation for the adverse health effects of air pollution. Fourthly, human-based evidence on specific particle size ranges, chemical compositions, and sources are still preliminary, and investigations based on large-scale prospective cohorts and use of more advanced statistical approaches are particularly helpful. Fifthly, there is a lack of clear understanding, especially from an exposomics perspective, on how external PM or its constituents enter the body, induce systemic biological functions, and ultimately result in a disease. Finally, the finer spatiotemporal resolution for exposure assessment, especially the personal exposure measurement or modeling, should be encouraged to improve the precision of estimated associations with health outcomes.

The coronavirus disease 2019 (COVID-19) pandemic has become a major health crisis worldwide. PM_2.5_ may not only act as a vital carrier and promote the persistence of the virus in the air, but it could also decrease immune response and increase susceptibility of viral infection. Recent epidemiological studies have shown that both short-term and long-term exposure to PM_2.5_ are related to the increase in spread and lethality of COVID-19.^159^, ^160^, ^161^ However, these epidemiological studies were subject to apparent limitations that may potentially mask true associations. These limitations mainly include the ecological or cross-sectional nature in design, as well as the inability to control for some critical factors responsible for the spread and lethality of COVID-19 (e.g., sociodemographic characteristics, control policy, population density, and the healthcare system capacity). On the other hand, while air quality during the lockdown period improved and the subsequent health benefits have been assessed,^162^ these associations were rarely examined in an epidemiological study. Therefore, the interactions of particulate air pollution and the COVID-19 pandemic warrant further investigations to better address the two pressing public health crises.

Climate change is widely considered the largest global public health challenge. The sources emitting greenhouse gases can also produce air pollutants, and they can interact with air pollution in inducing health effects. Accordingly, climate mitigation policies could also result in health co-benefits by virtue of reducing air pollution. As the largest emitter of carbon dioxide, China announced its ambitious climate commitment in September 2020 to achieve carbon peak by 2030 and to achieve carbon neutrality by 2060. The ambitious “double carbon” goal is a powerful driver for improving air quality in China. It was estimated that if China achieves the carbon neutrality goals in 2060, the annual average PM_2.5_ level (30 μg/m^3^ in 2021) will be reduced to about 8 μg/m^3^, which is very close to the new WHO AQG target (5 μg/m^3^), and approximately 78% of the Chinese population will experience an annual average PM_2.5_ level below 10 μg/m^3^.^163^ Air quality improvement actions aiming to reduce fossil fuel consumption will also promote low-carbon development. The achievement of carbon neutrality and the reduction of air pollution will in turn lead to considerable health co-benefits. Therefore, air quality, climate change, and health effects should be considered simultaneously to maximize public health benefits.

## Lead contact website

Renjie Chen: http://sph.fudan.edu.cn/employee/60

Haidong Kan: http://sph.fudan.edu.cn/employee/14

## References

[1] GBD 2019 Risk Factors Collaborators (2020). Global burden of 87 risk factors in 204 countries and territories, 1990-2019: a systematic analysis for the global burden of disease study 2019. Lancet.

[2] Xue T., Geng G., Meng X. (2022). New who global air quality guidelines help prevent premature deaths in China. Natl. Sci. Rev..

[3] Kan H., Chen R., Tong S. (2012). Ambient air pollution, climate change, and population health in China. Environ. Int..

[4] Health Effects Institute (2020).

[5] Yin P., He G., Fan M. (2017). Particulate air pollution and mortality in 38 of China's largest cities: time series analysis. BMJ.

[6] Sun Y., Zhang Y., Chen C. (2022). Impact of heavy PM(2.5) pollution events on mortality in 250 Chinese counties. Environ. Sci. Technol..

[7] Chen R., Yin P., Meng X. (2017). Fine particulate air pollution and daily mortality. A nationwide analysis in 272 Chinese cities. Am. J. Respir. Crit. Care Med..

[8] Dong Z., Wang H., Yin P. (2020). Time-weighted average of fine particulate matter exposure and cause-specific mortality in China: a nationwide analysis. Lancet Planet. Health.

[9] Atkinson R.W., Kang S., Anderson H.R. (2014). Epidemiological time series studies of pm2.5 and daily mortality and hospital admissions: a systematic review and meta-analysis. Thorax.

[10] He C., Liu C., Chen R. (2022). Fine particulate matter air pollution and under-5 children mortality in China: a national time-stratified case-crossover study. Environ. Int..

[11] Qiu H., Wang L., Zhou L., Pan J. (2020). Coarse particles (PM(2.5-10)) and cause-specific hospitalizations in southwestern China: association, attributable risk and economic costs. Environ. Res..

[12] Gu J., Shi Y., Zhu Y. (2020). Ambient air pollution and cause-specific risk of hospital admission in China: a nationwide time-series study. PLoS Med..

[13] Gu J., Shi Y., Chen N. (2020). Ambient fine particulate matter and hospital admissions for ischemic and hemorrhagic strokes and transient ischemic attack in 248 Chinese cities. Sci. Total Environ..

[14] Tian Y., Liu H., Wu Y. (2019). Association between ambient fine particulate pollution and hospital admissions for cause specific cardiovascular disease: time series study in 184 major Chinese cities. BMJ.

[15] Gu X., Guo T., Si Y. (2020). Association between ambient air pollution and daily hospital admissions for depression in 75 Chinese cities. Am. J. Psychiatry.

[16] Wang F., Liu H., Li H. (2018). Ambient concentrations of particulate matter and hospitalization for depression in 26 Chinese cities: a case-crossover study. Environ. Int..

[17] Ma Y., Wang W., Li Z. (2022). Short-term exposure to ambient air pollution and risk of daily hospital admissions for anxiety in China: a multicity study. J. Hazard Mater..

[18] Liang Z., Xu C., Ji A.L. (2020). Effects of short-term ambient air pollution exposure on hpv infections: a five-year hospital-based study. Chemosphere.

[19] Liang Z., Xu C., Fan Y.N. (2020). Association between air pollution and menstrual disorder outpatient visits: a time-series analysis. Ecotoxicol. Environ. Saf..

[20] Wu Y., Li H., Xu D. (2021). Associations of fine particulate matter and its constituents with airway inflammation, lung function, and buccal mucosa microbiota in children. Sci. Total Environ..

[21] Wang C., Cai J., Chen R. (2017). Personal exposure to fine particulate matter, lung function and serum club cell secretory protein (clara). Environ. Pollut..

[22] Mu G., Zhou M., Wang B. (2021). Personal PM(2.5) exposure and lung function: potential mediating role of systematic inflammation and oxidative damage in urban adults from the general population. Sci. Total Environ..

[23] Shi J., Chen R., Yang C. (2016). Association between fine particulate matter chemical constituents and airway inflammation: a panel study among healthy adults in China. Environ. Res..

[24] Fang J., Tang S., Zhou J. (2020). Associations between personal PM(2.5) elemental constituents and decline of kidney function in older individuals: the China bape study. Environ. Sci. Technol..

[25] Liu M., Guo W., Zhao L. (2021). Association of personal fine particulate matter and its respiratory tract depositions with blood pressure in children: from two panel studies. J. Hazard Mater..

[26] Zhou L., Tao Y., Li H. (2021). Acute effects of fine particulate matter constituents on cardiopulmonary function in a panel of COPD patients. Sci. Total Environ..

[27] Duan R., Niu H., Yu T. (2021). Adverse effects of short-term personal exposure to fine particulate matter on the lung function of patients with chronic obstructive pulmonary disease and asthma: a longitudinal panel study in beijing, China. Environ. Sci. Pollut. Res. Int..

[28] Chen S., Gu Y., Qiao L. (2017). Fine particulate constituents and lung dysfunction: a time-series panel study. Environ. Sci. Technol..

[29] Chi R., Chen C., Li H. (2019). Different health effects of indoor- and outdoor-originated PM(2.5) on cardiopulmonary function in copd patients and healthy elderly adults. Indoor Air.

[30] Lin Z., Niu Y., Chen R. (2017). Fine particulate matter constituents and blood pressure in patients with chronic obstructive pulmonary disease: a panel study in shanghai, China. Environ. Res..

[31] Lin Z., Wang X., Liu F. (2021). Impacts of short-term fine particulate matter exposure on blood pressure were modified by control status and treatment in hypertensive patients. Hypertension.

[32] Chen R., Qiao L., Li H. (2015). Fine particulate matter constituents, nitric oxide synthase DNA methylation and exhaled nitric oxide. Environ. Sci. Technol..

[33] Li H., Cai J., Chen R. (2017). Particulate matter exposure and stress hormone levels: a randomized, double-blind, crossover trial of air purification. Circulation.

[34] Chen R., Zhao A., Chen H. (2015). Cardiopulmonary benefits of reducing indoor particles of outdoor origin: a randomized, double-blind crossover trial of air purifiers. J. Am. Coll. Cardiol..

[35] Yang X., Jia X., Dong W. (2018). Cardiovascular benefits of reducing personal exposure to traffic-related noise and particulate air pollution: a randomized crossover study in the beijing subway system. Indoor Air.

[36] Shi J., Lin Z., Chen R. (2017). Cardiovascular benefits of wearing particulate-filtering respirators: a randomized crossover trial. Environ. Health Perspect..

[37] Gong J., Zhu T., Kipen H. (2014). Comparisons of ultrafine and fine particles in their associations with biomarkers reflecting physiological pathways. Environ. Sci. Technol..

[38] Yang F., Tan J., Zhao Q. (2011). Characteristics of pm2.5 speciation in representative megacities and across China. Atmos. Chem. Phys..

[39] Thurston G.D., Burnett R.T., Turner M.C. (2016). Ischemic heart disease mortality and long-term exposure to source-related components of u.S. Fine particle air pollution. Environ. Health Perspect..

[40] Yin P., Brauer M., Cohen A. (2017). Long-term fine particulate matter exposure and nonaccidental and cause-specific mortality in a large national cohort of Chinese men. Environ. Health Perspect..

[41] Yang X., Liang F., Li J. (2020). Associations of long-term exposure to ambient PM(2.5) with mortality in Chinese adults: a pooled analysis of cohorts in the China-par project. Environ. Int..

[42] Liang F., Liu F., Huang K. (2020). Long-term exposure to fine particulate matter and cardiovascular disease in China. J. Am. Coll. Cardiol..

[43] Li T., Zhang Y., Wang J. (2018). All-cause mortality risk associated with long-term exposure to ambient PM(2·5) in China: a cohort study. Lancet Public Health.

[44] Li J., Lu X., Liu F. (2020). Chronic effects of high fine particulate matter exposure on lung cancer in China. Am. J. Respir. Crit. Care Med..

[45] Yang X., Zhang L., Chen X. (2021). Long-term exposure to ambient PM(2.5) and stroke mortality among urban residents in northern China. Ecotoxicol. Environ. Saf..

[46] Ran J., Yang A., Sun S. (2020). Long-term exposure to ambient fine particulate matter and mortality from renal failure: a retrospective cohort study in Hong Kong, China. Am. J. Epidemiol..

[47] Lepeule J., Laden F., Dockery D., Schwartz J. (2012). Chronic exposure to fine particles and mortality: an extended follow-up of the harvard six cities study from 1974 to 2009. Environ. Health Perspect..

[48] Beelen R., Raaschou-Nielsen O., Stafoggia M. (2014). Effects of long-term exposure to air pollution on natural-cause mortality: an analysis of 22 european cohorts within the multicentre escape project. Lancet.

[49] Liu S., Zhou Y., Liu S. (2017). Association between exposure to ambient particulate matter and chronic obstructive pulmonary disease: results from a cross-sectional study in China. Thorax.

[50] Wang C., Xu J., Yang L., China Pulmonary Health Study Group (2018). Prevalence and risk factors of chronic obstructive pulmonary disease in China (the China pulmonary health [cph] study): a national cross-sectional study. Lancet.

[51] Li J., Liu F., Liang F. (2020). Long-term effects of high exposure to ambient fine particulate matter on coronary heart disease incidence: a population-based Chinese cohort study. Environ. Sci. Technol..

[52] Huang K., Liang F., Yang X. (2019). Long term exposure to ambient fine particulate matter and incidence of stroke: prospective cohort study from the China-par project. BMJ.

[53] Liu L., Zhang Y., Yang Z. (2021). Long-term exposure to fine particulate constituents and cardiovascular diseases in Chinese adults. J. Hazard Mater..

[54] Guo Y., Zeng H., Zheng R. (2016). The association between lung cancer incidence and ambient air pollution in China: a spatiotemporal analysis. Environ. Res..

[55] Zhang Z., Zhu D., Cui B. (2020). Association between particulate matter air pollution and lung cancer. Thorax.

[56] Liang F., Yang X., Liu F. (2019). Long-term exposure to ambient fine particulate matter and incidence of diabetes in China: a cohort study. Environ. Int..

[57] Huang S., Zhang X., Liu Z. (2021). Long-term impacts of ambient fine particulate matter exposure on overweight or obesity in Chinese adults: the China-par project. Environ. Res..

[58] Huang K., Yang X., Liang F. (2019). Long-term exposure to fine particulate matter and hypertension incidence in China. Hypertension.

[59] Liu C., Yang C., Zhao Y. (2016). Associations between long-term exposure to ambient particulate air pollution and type 2 diabetes prevalence, blood glucose and glycosylated hemoglobin levels in China. Environ. Int..

[60] Yang B.Y., Qian Z.M., Li S. (2018). Ambient air pollution in relation to diabetes and glucose-homoeostasis markers in China: a cross-sectional study with findings from the 33 communities Chinese health study. Lancet Planet. Health.

[61] Li Q., Zheng D., Wang Y. (2021). Association between exposure to airborne particulate matter less than 2.5 μm and human fecundity in China. Environ. Int..

[62] Qiu Y., Yang T., Seyler B.C. (2020). Ambient air pollution and male fecundity: a retrospective analysis of longitudinal data from a Chinese human sperm bank (2013-2018). Environ. Res..

[63] Chen Q., Wang F., Yang H. (2021). Exposure to fine particulate matter-bound polycyclic aromatic hydrocarbons, male semen quality, and reproductive hormones: the marchs study. Environ. Pollut..

[64] Zhang Y., Li J., Liao J. (2021). Impacts of ambient fine particulate matter on blood pressure pattern and hypertensive disorders of pregnancy: evidence from the wuhan cohort study. Hypertension.

[65] Ye B., Zhong C., Li Q. (2020). The associations of ambient fine particulate matter exposure during pregnancy with blood glucose levels and gestational diabetes mellitus risk: a prospective cohort study in wuhan, China. Am. J. Epidemiol..

[66] Li Q., Wang Y.Y., Guo Y. (2018). Effect of airborne particulate matter of 2.5 μm or less on preterm birth: a national birth cohort study in China. Environ. Int..

[67] Zhang J., Chen G., Liang S. (2021). PM(2.5) exposure exaggerates the risk of adverse birth outcomes in pregnant women with pre-existing hyperlipidemia: modulation role of adipokines and lipidome. Sci. Total Environ..

[68] Liu A., Qian N., Yu H. (2017). Estimation of disease burdens on preterm births and low birth weights attributable to maternal fine particulate matter exposure in shanghai, China. Sci. Total Environ..

[69] Lin L., Li Q., Yang J. (2020). The associations of particulate matters with fetal growth in utero and birth weight: a birth cohort study in beijing, China. Sci. Total Environ..

[70] Han Y., Wang W., Wang X. (2020). Prenatal exposure to fine particles, premature rupture of membranes and gestational age: a prospective cohort study. Environ. Int..

[71] Yang B.Y., Qu Y., Guo Y. (2021). Maternal exposure to ambient air pollution and congenital heart defects in China. Environ. Int..

[72] Liang Z., Yang Y., Yi J. (2021). Maternal PM(2.5) exposure associated with stillbirth: a large birth cohort study in seven Chinese cities. Int. J. Hyg Environ. Health.

[73] Jung C.R., Chen W.T., Tang Y.H., Hwang B.F. (2019). Fine particulate matter exposure during pregnancy and infancy and incident asthma. J. Allergy Clin. Immunol..

[74] Mao G., Nachman R.M., Sun Q. (2017). Individual and joint effects of early-life ambient exposure and maternal prepregnancy obesity on childhood overweight or obesity. Environ. Health Perspect..

[75] Chen G., Jin Z., Li S. (2018). Early life exposure to particulate matter air pollution (PM(1), PM(2.5) and PM(10)) and autism in shanghai, China: a case-control study. Environ. Int..

[76] Wang P., Zhao Y., Li J. (2021). Prenatal exposure to ambient fine particulate matter and early childhood neurodevelopment: a population-based birth cohort study. Sci. Total Environ..

[77] Zhou Y., Ma J., Wang B. (2020). Long-term effect of personal PM(2.5) exposure on lung function: a panel study in China. J. Hazard Mater..

[78] Yang T., Chen R., Gu X., China Pulmonary Health Study Group (2021). Association of fine particulate matter air pollution and its constituents with lung function: the China pulmonary health study. Environ. Int..

[79] Lin H., Guo Y., Zheng Y. (2017). Long-term effects of ambient PM(2.5) on hypertension and blood pressure and attributable risk among older Chinese adults. Hypertension.

[80] Zhang Z., Dong B., Li S. (2019). Exposure to ambient particulate matter air pollution, blood pressure and hypertension in children and adolescents: a national cross-sectional study in China. Environ. Int..

[81] Sun D., Liu Y., Zhang J. (2021). Long-term effects of fine particulate matter exposure on the progression of arterial stiffness. Environ. Health..

[82] Li Q., Wang Y.Y., Guo Y. (2021). Association between airborne particulate matter and renal function: an analysis of 2.5 million young adults. Environ. Int..

[83] Wang J., Li T., Lv Y. (2020). Fine particulate matter and poor cognitive function among Chinese older adults: evidence from a community-based, 12-year prospective cohort study. Environ. Health Perspect..

[84] Zhang Z., Dong B., Li S. (2019). Particulate matter air pollution and blood glucose in children and adolescents: a cross-sectional study in China. Sci. Total Environ..

[85] Mao S., Chen G., Liu F. (2020). Long-term effects of ambient air pollutants to blood lipids and dyslipidemias in a Chinese rural population. Environ. Pollut..

[86] Brook R.D., Sun Z., Brook J.R. (2016). Extreme air pollution conditions adversely affect blood pressure and insulin resistance: the air pollution and cardiometabolic disease study. Hypertension.

[87] Zhang Z., Chan T.C., Guo C. (2018). Long-term exposure to ambient particulate matter (PM(2.5)) is associated with platelet counts in adults. Environ. Pollut..

[88] Li Z., Yan H., Zhang X. (2021). Air pollution interacts with genetic risk to influence cortical networks implicated in depression. Proc. Natl. Acad. Sci. US..

[89] HEI Accountability Working Group (2003). Communication 11.

[90] Xue T., Guan T., Zheng Y. (2021). Long-term PM(2.5) exposure and depressive symptoms in China: a quasi-experimental study. Lancet Reg. Health. West. Pac..

[91] Li J., Yao Y., Xie W. (2021). Association of long-term exposure to PM(2.5) with blood lipids in the Chinese population: findings from a longitudinal quasi-experiment. Environ. Int..

[92] Xue T., Zhu T., Peng W. (2021). Clean air actions in China, pm2.5 exposure, and household medical expenditures: a quasi-experimental study. PLoS Med..

[93] Yao Y., Lv X., Qiu C. (2022). The effect of China's clean air act on cognitive function in older adults: a population-based, quasi-experimental study. Lancet. Healthy Longev..

[94] Wang W., Primbs T., Tao S., Simonich S.L.M. (2009). Atmospheric particulate matter pollution during the 2008 beijing olympics. Environ. Sci. Technol..

[95] Su C., Hampel R., Franck U. (2015). Assessing responses of cardiovascular mortality to particulate matter air pollution for pre-during- and post-2008 olympics periods. Environ. Res..

[96] Rich D.Q., Liu K., Zhang J. (2015). Differences in birth weight associated with the 2008 beijing olympics air pollution reduction: results from a natural experiment. Environ. Health Perspect..

[97] Rich D.Q., Kipen H.M., Huang W. (2012). Association between changes in air pollution levels during the beijing olympics and biomarkers of inflammation and thrombosis in healthy young adults. JAMA.

[98] Huang W., Wang G., Lu S.E. (2012). Inflammatory and oxidative stress responses of healthy young adults to changes in air quality during the beijing olympics. Am. J. Respir. Crit. Care Med..

[99] Lin W., Zhu T., Xue T. (2015). Association between changes in exposure to air pollution and biomarkers of oxidative stress in children before and during the beijing olympics. Am. J. Epidemiol..

[100] Lin W., Huang W., Zhu T. (2011). Acute respiratory inflammation in children and black carbon in ambient air before and during the 2008 beijing olympics. Environ. Health Perspect..

[101] Li H., Zhou L., Wang C. (2017). Associations between air quality changes and biomarkers of systemic inflammation during the 2014 nanjing youth olympics: a quasi-experimental study. Am. J. Epidemiol..

[102] Lin Y., Ramanathan G., Zhu Y. (2019). Pro-oxidative and proinflammatory effects after traveling from los angeles to beijing: a biomarker-based natural experiment. Circulation.

[103] Wu S., Deng F., Hao Y. (2014). Fine particulate matter, temperature, and lung function in healthy adults: findings from the hvnr study. Chemosphere.

[104] Wu S., Deng F., Huang J. (2013). Blood pressure changes and chemical constituents of particulate air pollution: results from the healthy volunteer natural relocation (hvnr) study. Environ. Health Perspect..

[105] Wu S., Deng F., Wei H. (2014). Association of cardiopulmonary health effects with source-appointed ambient fine particulate in beijing, China: a combined analysis from the healthy volunteer natural relocation (hvnr) study. Environ. Sci. Technol..

[106] Chen R., Meng X., Zhao A. (2016). DNA hypomethylation and its mediation in the effects of fine particulate air pollution on cardiovascular biomarkers: a randomized crossover trial. Environ. Int..

[107] Chen R., Li H., Cai J. (2018). Fine particulate air pollution and the expression of micrornas and circulating cytokines relevant to inflammation, coagulation, and vasoconstriction. Environ. Health Perspect..

[108] Li H., Chen R., Cai J. (2018). Short-term exposure to fine particulate air pollution and genome-wide DNA methylation: a randomized, double-blind, crossover trial. Environ. Int..

[109] Guo M., Du C., Li B. (2021). Reducing particulates in indoor air can improve the circulation and cardiorespiratory health of old people: a randomized, double-blind crossover trial of air filtration. Sci. Total Environ..

[110] Shao D., Du Y., Liu S. (2017). Cardiorespiratory responses of air filtration: a randomized crossover intervention trial in seniors living in beijing: beijing indoor air purifier study, biapsy. Sci. Total Environ..

[111] Langrish J.P., Li X., Wang S. (2012). Reducing personal exposure to particulate air pollution improves cardiovascular health in patients with coronary heart disease. Environ. Health Perspect..

[112] Guan T., Hu S., Han Y. (2018). The effects of facemasks on airway inflammation and endothelial dysfunction in healthy young adults: a double-blind, randomized, controlled crossover study. Part. Part. Fibre Toxicol..

[113] Lin Z., Chen R., Jiang Y. (2019). Cardiovascular benefits of fish-oil supplementation against fine particulate air pollution in China. J. Am. Coll. Cardiol..

[114] Lin Z., Niu Y., Jiang Y. (2021). Protective effects of dietary fish-oil supplementation on skin inflammatory and oxidative stress biomarkers induced by fine particulate air pollution: a pilot randomized, double-blind, placebo-controlled trial. Br. J. Dermatol..

[115] Li H., Liu Q., Zou Z. (2021). L-arginine supplementation to mitigate cardiovascular effects of walking outside in the context of traffic-related air pollution in participants with elevated blood pressure: a randomized, double-blind, placebo-controlled trial. Environ. Int..

[116] Liu Q., Li H., Guo L. (2021). Effects of short-term personal exposure to air pollution on platelet mitochondrial DNA methylation levels and the potential mitigation by l-arginine supplementation. J. Hazard Mater..

[117] Wu S., Deng F., Hao Y. (2013). Chemical constituents of fine particulate air pollution and pulmonary function in healthy adults: the healthy volunteer natural relocation study. J. Hazard Mater..

[118] Wu S., Yang D., Pan L. (2016). Chemical constituents and sources of ambient particulate air pollution and biomarkers of endothelial function in a panel of healthy adults in beijing, China. Sci. Total Environ..

[119] Wu S., Yang D., Wei H. (2015). Association of chemical constituents and pollution sources of ambient fine particulate air pollution and biomarkers of oxidative stress associated with atherosclerosis: a panel study among young adults in beijing, China. Chemosphere.

[120] Liang R., Chen R., Yin P. (2022). Associations of long-term exposure to fine particulate matter and its constituents with cardiovascular mortality: a prospective cohort study in China. Environ. Int..

[121] He Y., Jiang Y., Yang Y. (2022). Composition of fine particulate matter and risk of preterm birth: a nationwide birth cohort study in 336 Chinese cities. J. Hazard Mater..

[122] Chen L., Zhang Y., Zhang W. (2020). Short-term effect of PM(1) on hospital admission for ischemic stroke: a multi-city case-crossover study in China. Environ. Pollut..

[123] Lin H., Tao J., Du Y. (2016). Particle size and chemical constituents of ambient particulate pollution associated with cardiovascular mortality in guangzhou, China. Environ. Pollut..

[124] Wu Q.Z., Li S., Yang B.Y. (2020). Ambient airborne particulates of diameter ≤1 μm, a leading contributor to the association between ambient airborne particulates of diameter ≤2.5 μm and children's blood pressure. Hypertension.

[125] Yang M., Chu C., Bloom M.S. (2018). Is smaller worse? New insights about associations of PM(1) and respiratory health in children and adolescents. Environ. Int..

[126] Wang Y.Y., Li Q., Guo Y. (2018). Association of long-term exposure to airborne particulate matter of 1 μm or less with preterm birth in China. JAMA Pediatr..

[127] Zhang Z., Dong B., Chen G. (2021). Ambient air pollution and obesity in school-aged children and adolescents: a multicenter study in China. Sci. Total Environ..

[128] Chen G., Li S., Zhang Y. (2017). Effects of ambient PM(1) air pollution on daily emergency hospital visits in China: an epidemiological study. Lancet Planet. Health.

[129] Li H., Li X., Zheng H. (2021). Ultrafine particulate air pollution and pediatric emergency-department visits for main respiratory diseases in shanghai, China. Sci. Total Environ..

[130] Guo P.Y., He Z.Z., Jalaludin B. (2021). Short-term effects of particle size and constituents on blood pressure in healthy young adults in guangzhou, China. J. Am. Heart Assoc..

[131] He Z.Z., Guo P.Y., Xu S.L. (2021). Associations of particulate matter sizes and chemical constituents with blood lipids: a panel study in guangzhou, China. Environ. Sci. Technol..

[132] Huang C., Tang M., Li H. (2021). Particulate matter air pollution and reduced heart rate variability: how the associations vary by particle size in shanghai, China. Ecotoxicol. Environ. Saf..

[133] Feng D., Cao K., He Z.Z. (2021). Short-term effects of particle sizes and constituents on blood biomarkers among healthy young adults in guangzhou, China. Environ. Sci. Technol..

[134] (2021). Particulate Matter (PM2.5 and PM10), Ozone, Nitrogen Dioxide, Sulfur Dioxide and Carbon Monoxide.

[135] Chuang H.C., Ho K.F., Lin L.Y. (2017). Long-term indoor air conditioner filtration and cardiovascular health: a randomized crossover intervention study. Environ. Int..

[136] Zhao Y., Xue L., Chen Q. (2020). Cardiorespiratory responses to fine particles during ambient PM(2.5) pollution waves: findings from a randomized crossover trial in young healthy adults. Environ. Int..

[137] Chen X., Han Y., Chen W. (2020). Respiratory inflammation and short-term ambient air pollution exposures in adult beijing residents with and without prediabetes: a panel study. Environ. Health Perspect..

[138] Yao Y., Chen X., Chen W. (2021). Susceptibility of individuals with chronic obstructive pulmonary disease to respiratory inflammation associated with short-term exposure to ambient air pollution: a panel study in beijing. Sci. Total Environ..

[139] Zhang Q., Wang W., Niu Y. (2019). The effects of fine particulate matter constituents on exhaled nitric oxide and DNA methylation in the arginase-nitric oxide synthase pathway. Environ. Int..

[140] Xia B., Zhou Y., Zhu Q. (2019). Personal exposure to PM(2.5) constituents associated with gestational blood pressure and endothelial dysfunction. Environ. Pollut..

[141] Hu D., Jia X., Cui L. (2021). Exposure to fine particulate matter promotes platelet activation and thrombosis via obesity-related inflammation. J. Hazard Mater..

[142] Xu H., Wang T., Liu S. (2019). Extreme levels of air pollution associated with changes in biomarkers of atherosclerotic plaque vulnerability and thrombogenicity in healthy adults. Circ. Res..

[143] Wang C., Chen R., Shi M. (2018). Possible mediation by methylation in acute inflammation following personal exposure to fine particulate air pollution. Am. J. Epidemiol..

[144] Li J., Wang T., Wang Y. (2020). Particulate matter air pollution and the expression of micrornas and pro-inflammatory genes: association and mediation among children in jinan, China. J. Hazard Mater..

[145] Zhong J., Karlsson O., Wang G. (2017). B vitamins attenuate the epigenetic effects of ambient fine particles in a pilot human intervention trial. Proc. Natl. Acad. Sci. USA.

[146] Wang M., Zhao J., Wang Y. (2020). Genome-wide DNA methylation analysis reveals significant impact of long-term ambient air pollution exposure on biological functions related to mitochondria and immune response. Environ. Pollut..

[147] Huang Q., Hu D., Wang X. (2018). The modification of indoor PM(2.5) exposure to chronic obstructive pulmonary disease in Chinese elderly people: a meet-in-metabolite analysis. Environ. Int..

[148] Mu L., Niu Z., Blair R.H. (2019). Metabolomics profiling before, during, and after the beijing olympics: a panel study of within-individual differences during periods of high and low air pollution. Environ. Health Perspect..

[149] Chen C., Li H., Niu Y. (2019). Impact of short-term exposure to fine particulate matter air pollution on urinary metabolome: a randomized, double-blind, crossover trial. Environ. Int..

[150] Zhang Y., Chu M., Zhang J. (2019). Urine metabolites associated with cardiovascular effects from exposure of size-fractioned particulate matter in a subway environment: a randomized crossover study. Environ. Int..

[151] Zheng P., Zhang B., Zhang K. (2020). The impact of air pollution on intestinal microbiome of asthmatic children: a panel study. BioMed Res. Int..

[152] Liu T., Chen X., Xu Y. (2019). Gut microbiota partially mediates the effects of fine particulate matter on type 2 diabetes: evidence from a population-based epidemiological study. Environ. Int..

[153] Li H., Xu D., Li H. (2021). Exposure to ultrafine particles and oral flora, respiratory function, and biomarkers of inflammation: a panel study in children. Environ. Pollut..

[154] Wang L., Cheng H., Wang D. (2019). Airway microbiome is associated with respiratory functions and responses to ambient particulate matter exposure. Ecotoxicol. Environ. Saf..

[155] Li X., Sun Y., An Y. (2019). Air pollution during the winter period and respiratory tract microbial imbalance in a healthy young population in northeastern China. Environ. Pollut..

[156] Yao Y., Chen X., Chen W. (2021). Differences in transcriptome response to air pollution exposure between adult residents with and without chronic obstructive pulmonary disease in beijing: a panel study. J. Hazard Mater..

[157] Du X., Zhang Q., Jiang Y., Li H., Zhu X., Zhang Y., Liu C., Niu Y., Ji J., Jiang C., Cai J., Chen R., Kan H. (2022). Dynamic molecular choreography induced by traffic exposure: a randomized, crossover trial using multi-omics profiling. J. Hazard Mater..

[158] Zhang Q., Du X., Li H. (2022). Cardiovascular effects of traffic-related air pollution: a multi-omics analysis from a randomized, crossover trial. J. Hazard Mater..

[159] Copat C., Cristaldi A., Fiore M. (2020). The role of air pollution (PM and no(2)) in COVID-19 spread and lethality: a systematic review. Environ. Res..

[160] Tian F., Liu X., Chao Q. (2021). Ambient air pollution and low temperature associated with case fatality of COVID-19: a nationwide retrospective cohort study in China. Innovation.

[161] Xing X., Xiong Y., Yang R. (2021). Predicting the effect of confinement on the COVID-19 spread using machine learning enriched with satellite air pollution observations. Proc. Natl. Acad. Sci. USA.

[162] Chen K., Wang M., Huang C. (2020). Air pollution reduction and mortality benefit during the COVID-19 outbreak in China. Lancet Planet. Health.

[163] Cheng J., Tong D., Zhang Q. (2021). Pathways of China's PM(2.5) air quality 2015-2060 in the context of carbon neutrality. Natl. Sci. Rev..

